# Spin effect regulation as a design principle for M–N–C catalysts for oxygen electrocatalysis

**DOI:** 10.1039/d6sc00439c

**Published:** 2026-03-30

**Authors:** Haiyan Li, Zhanzhao Fu, Yuan Yuan, Di Zhang, Yubo Chen, Hao Li, Ang Cao

**Affiliations:** a State Key Laboratory of Clean Energy Utilization, College of Energy Engineering, Zhejiang University Hangzhou 310027 China li.hao.b8@tohoku.ac.jp angc@zju.edu.cn; b Key Laboratory of Biomass Chemical Engineering of Ministry of Education, College of Chemical and Biological Engineering, Zhejiang University Hangzhou 310027 China; c Hydrogen Energy Institute, Zhejiang University Hangzhou 310027 China; d Suzhou MatSource Technology Co., Ltd. Suzhou 215000 China; e Advanced Institute for Materials Research (WPI-AIMR), Tohoku University Sendai 980-8577 Japan; f Institute of Advanced Equipment, College of Energy Engineering, Zhejiang University Hangzhou 310027 China; g Inner Mongolia Daqingshan Laboratory Hohhot 017000 China

## Abstract

Recent advances indicate that regulating the spin state of metal centers can markedly modulate catalytic performance, motivating interest in exploiting spin effects for the design of highly efficient oxygen electrocatalysts. However, the fundamental mechanisms linking spin state and activity remain insufficiently understood. Herein, M–N–C catalysts (M = Fe/Co/Ni) with atomically well-defined coordination environments are employed to systematically analyze spin effects on oxygen electrocatalysis, enabling precise control of the metal-center spin state *via* local coordination tuning. By means of density functional theory calculations and state-of-the-art microkinetic volcano modeling, we show clear scaling correlations between the spin moment of metal center and the adsorption energies of key intermediates (*i.e.*, HO*, O*, and HOO*) of Fe/Co/Ni single-atom sites, which in turn dictate activity trends in oxygen evolution and reduction reaction (ORR and OER) activities through the modulation of the metal-center spin. Interestingly, optimal spin moments of 0.5, 1.0, and 0.65 *µ*_B_ correspond to OER, ORR, and bifunctional oxygen electrocatalysis, respectively. Leveraging the spin moment as activity descriptors, we rapidly screened spin states of Fe/Co/Ni sites on three dual-atom M–N–C frameworks and predicted highly active oxygen electrocatalysts, subsequently validated by DFT calculations. These findings establish a rapid, quantitative, spin effect-based design principle for rational development and accelerated discovery of high-performance oxygen electrocatalysts.

## Abstract 2

Recent advances indicate that regulating the spin state of metal centers can markedly modulate catalytic performance, motivating interest in exploiting spin effects for the design of highly efficient oxygen electrocatalysts.

## Graphical abstract

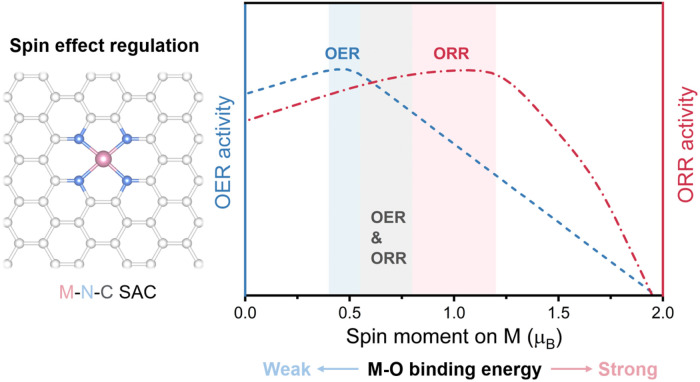

## 1 Introduction

Oxygen electrocatalysis is a cornerstone for renewable energy conversion and storage technologies, including water electrotysers, fuel cells, and metal–air batteries.^1–3^ However, the large intrinsic overpotentials associated with the oxygen evolution reaction (OER) and oxygen reduction reaction (ORR) significantly limit the overall efficiency of these devices.^4^ Over the past decades, considerable effort has therefore been devoted to discovering high-performance electrocatalysts to accelerate the OER/ORR kinetics. Although Ir- and Pt-based catalysts exhibit outstanding activity for OER and ORR, respectively, the high cost and limited availability of noble metals makes developing alternatives based on earth-abundant elements a priority.^5^ In this context, catalysts based on first-row transition metal (TM) have emerged as promising candidates owing to their low cost and commendable activity. Moreover, compared with noble metals, 3d TMs are characterized by relatively lower crystal field splitting energies and more readily formed open-shell configurations, exhibiting a wider variety of oxidation/spin states and electronic/magnetic properties.

Recently, growing research attention has focused on understanding the fundamental correlation between the spin configurations of 3d TM centers (particularly Fe, Co, and Ni) and their OER/ORR catalytic performance.^6–12^ A comprehensive understanding of how exactly spin states govern catalytic activity remains elusive. Additionally, due to the difficulty of precisely controlling and experimentally characterizing the spin states of catalyst surfaces, the development of spin-related design principles to guide the screening of electrocatalysts is still in its infancy.^13^

Heterogeneous single-atom metal–nitrogen–carbon catalysts (namely, M–N–C SACs), featuring isolated metal atoms (*e.g*., magnetic elements) anchored on conductive substrates such as carbon-based materials, are renowned for their exceptional catalytic efficiency and maximum atomic utilization. The electronic structures of the well-defined metal active centers can be meticulously controlled with multiple strategies.^14–18^ In particular, the spin configurations of the central magnetic metal atoms can be finely tuned by engineering their local coordination environments, offering a direct pathway to alter their intrinsic catalytic performance.^18–22^ Such precise control makes SACs an ideal platform for investigating the intrinsic structure–performance relationships in spin catalysts, as suggested by previous works that the on-site magnetic moment can serve as an activity descriptor for the ORR on Fe-/Co-centered SACs.^23–27^ However, the broader applicability of this spin-related descriptor to other metals such as Ni or to other reactions such as the OER, as well as quantitative design guidance such as optimal spin-moment values for catalyst improvement, remain to be further explored. Beyond the thermodynamic analyses of spin effects based on limiting potentials as activity metrics,^27–29^ incorporating microkinetic modeling may provide additional insights into the role of metal spin moments in the OER and ORR activity on M–N–C SACs.

To deepen the understanding of spin effects in oxygen electrocatalysis and to advance the rational design of electrocatalysts *via* a descriptor-based approach, single-atom Fe/Co/Ni embedded in N-doped graphene is used as a theoretical model in this study to explore the correlations between the spin state and the inherent activity for OER/ORR catalysis. By precisely regulating the spin moment on magnetic metal center through varying the number of coordinating N atom, DFT calculations reveal that the adsorption energies of oxygenated intermediates enhance linearly with the spin moment on Fe/Co/Ni sites. Based on the linear scaling relations among HO*, HO*, and HOO*, the OER/ORR activity (determined by the cutting-edge microkinetic modeling analyses) of these SACs can be solely described by the HO* adsorption energy and, consequently, by the spin moment. Accordingly, spin moment can serve as an effective descriptor not only for Fe-based but also for Co- and Ni-based SACs in oxygen electrocatalysis.

Guided by this insight, we propose following spin-engineering strategies: for strong-binding metal sites (*e.g*., Fe–N–C), activity can be optimized by reducing the spin moment; conversely, for weak-binding metal sites (*e.g*., the NiN_4_ site), activity can be enhanced by enlarging the spin moment. To further improve catalytic performance, we extend this principle to dual-atom M–N–C catalysts (DACs), where the spin moment of Fe/Co/Ni site is nuancedly regulated by incorporating a neighboring transition metal atom. With the guidance of magnetic moment-based descriptor and consideration of synthesis feasibility, promising DACs across three different structural configurations for bifunctional OER/ORR catalysis were quickly screened out. These findings demonstrate that regulating the spin moments of single-atom magnetic metal sites through coordination engineering is a powerful strategy for catalyst development. Most importantly, this work provides a clear framework and valuable guidance for the future rational design of spin-tailored catalysts with optimized multi-functional electrocatalytic activity.

## 2 Results and discussion

### 2.1. Spin-state regulation of single-atom sites by modifying the number of coordinating N

**Fig. 1:**
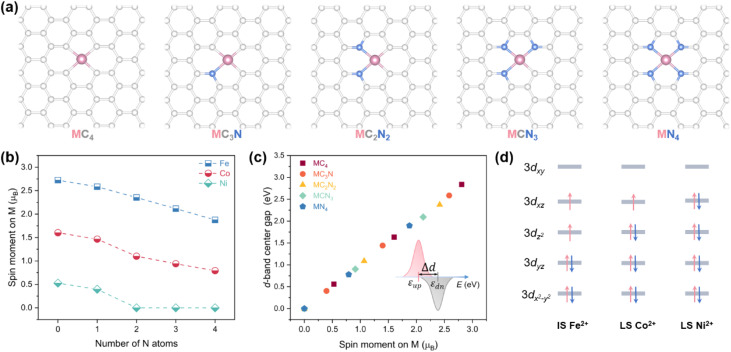
(a) Atomic models of M–N–C (M = Fe/Co/Ni) SACs. N and M atoms are colored blue and pink, respectively. (b) Magnetic moment of metal atom in M–N–C as a function of coordinating N atom number. (c) The d-band center gap (Δ*d*) of M atom as a function of the spin moment. (d) 3d electronic configurations of IS Fe^2+^ in FeN_4_, low-spin (LS) Co^2+^ in CoN_4_, and LS Ni^2+^ in NiN_4_ sites.

First, we systematically modulated the spin states of Fe, Co, and Ni centers by gradually reducing the number of N atoms within their first coordination shell (Fig. 1a). For the MC_2_N_2_ (M = Fe/Co/Ni) models, the most stable atomic configurations were selected for investigation (Fig. S1). As the coordinating nitrogen number decreases from four to zero, the spin magnetic moment of the central metal atom progressively increases (Fig. 1b and Table S1), which reflects a gradual increment in the number of unpaired d electrons. Specifically, the magnetic moment of Fe linearly increases from 1.88 to 2.72 *µ*_B_, that of Co from 0.79 to 1.60 *µ*_B_, and that of Ni from 0 to 0.52 *µ*_B_. To elucidate the changes in electronic structure, we analyzed the projected density of states (pDOSs) for the metal 3d orbitals in these M–N–C SACs (Fig. S2–S4). Orbital occupation was determined by integrating the DOS below the Fermi level. Due to electrostatic repulsion between the outer electrons of the central metal and ligands, the five initially degenerate 3d orbitals undergo energy splitting, resulting in the following energy ordering: 3d_*x*^2^*–y*^2^_ < 3d_*yz*_ < 3d_*z*^2^_ < 3d_*xz*_ < 3d_*xy*_.^23,30^ Electron filling in these split orbitals is governed by a balance between the spin-pairing energy and the ligand-filed splitting energy, and the latter scales with the strength of metal–ligand interaction.^13,31^ For example, according to the pDOS plot of the FeN_4_ site (Fig. S2a), its Fe center adopts an intermediate-spin (IS) Fe(ii) configuration, which is characterized by an empty 3d_*xy*_ orbital, singly occupied 3d_*xz*_ and 3d_*z*^2^_ orbitals, and fully occupied 3*d*_*yz*_ and 3d_*x*^2^*–y*^2^_ orbitals (Fig. 1d), yielding two unpaired electrons and spin magnetic moment of ∼2 *µ*_B_. With the reduction of N coordination, the amount of valence electrons on the Fe center remains essentially constant at six (Fig. S2f). Nonetheless, as C (2.55) atom has lower electronegativity than N (3.04),^22^ the electronegativity difference between the coordinating atoms weakens the electronic repulsive interactions between the Fe and its nearest-neighbor ligand atoms.^32^ This reduction in ligand-field strength allows the higher-energy 3d_*xy*_ orbital to become partially occupied, thereby increasing the number of unpaired d electrons. Analogously, upon a d^7^ Co^2+^ or d^8^ Ni^2+^ ion placed in the identical coordination environment as the d^6^ Fe^2+^ ion, the additional electrons preferentially fill the unoccupied spin-down orbitals, leading to fewer net unpaired d-electrons and lower spin moments (Fig. 1d, S3 and S4).

Meanwhile, the asymmetry between the spin-up and spin-down pDOS also varies consistently with the changes in spin moment. In N-rich coordination environments, this asymmetry is less pronounced, rendering a smaller separation between the d-band centers of the spin-up and spin-down channels (Fig. 1c). For instance, the d-band center gap of the FeC_4_ site is 2.84 eV, reflecting strong spin polarization, whereas that of the FeN_4_ site is reduced to 1.90 eV, which aligns with the spin moment difference of ∼1 *µ*_B_ between the two sites. Similar trends are observed for Co–N–C and Ni–N–C SACs, where the d-band center gap narrows linearly with the decrease in spin magnetic moment. For Ni sites with spin moment of 0 *µ*_B_, d-band center gap becomes zero, indicating no spin polarization.

The conventional mechanism for the OER on a single active site (denoted as *) proceeds through the sequential formation of intermediates HO*, O*, and HOO*, while the four-electron (4e^−^) ORR mechanism approximately follows the reverse pathway along the same intermediates. We note that the scaling relations and the subsequent microkinetic modeling are based on the adsorbate evolution mechanism (AEM), which is the prevailing pathway for M–N–C catalysts where the isolated metal center serves as the primary active site. Alternative pathways such as the lattice oxygen mechanism (LOM),^33^ typically observed in metal oxides with reactive lattice oxygen sites, are not considered in this work.

**Fig. 2:**
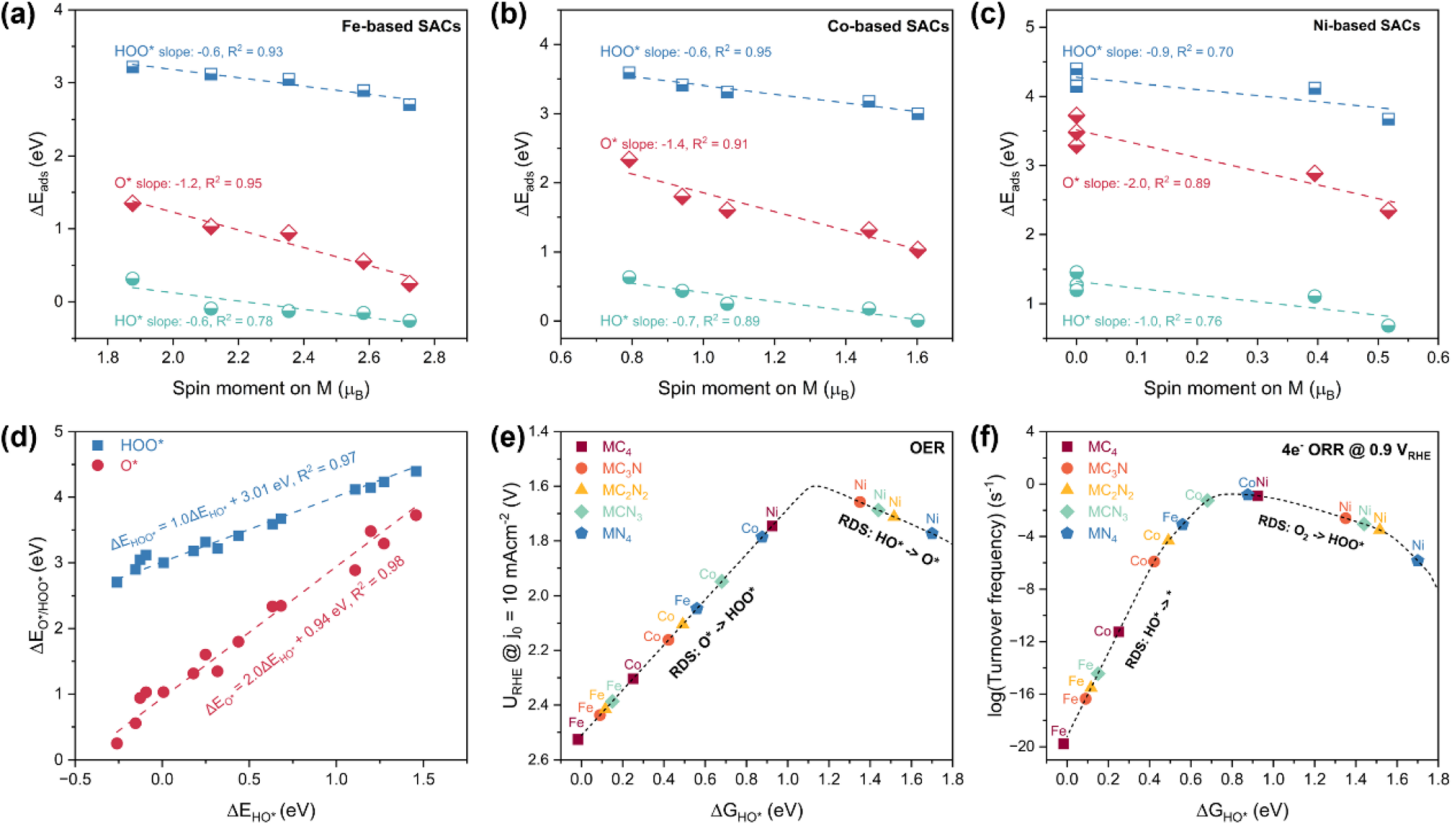
HO*, O*, and HOO* binding energies as a function of metal magnetic moment for (a) Fe, (b) Co, and (c) Ni-based SACs. The dashed lines and the values of slopes and coefficients of determination (*R*^2^) in (a–c) are obtained through linear fitting of the corresponding data. (d) Scaling relationships between O*/HOO* and HO* binding energies. Microkinetic modeling derived (e) OER and (f) ORR activities as a function of HO* adsorption free energy on the Fe/Co/Ni sites. The indicated OER/ORR volcano trends (black dashed lines in (e) and (f), respectively) are derived according to the linear scaling relations shown in (d) and microkinetic modeling analysis.

The adsorption strengths of these intermediates are primarily governed by the electronic interactions between the 3d orbital of the TM center and the 2p orbital of the adsorbed oxygen atom. Consequently, variations in the spin moment of the TM site, which reflect changes in its d-electron configuration, naturally influence the binding energies of these oxygenate intermediates (denoted as Δ*E*_HO*_, Δ*E*_O*_, and Δ*E*_HOO*_, respectively). For either Fe, Co, or Ni sites, as the spin moment increases, the adsorption free energies all monotonically decrease, corresponding to a strengthening of binding (Fig. 2a–c). For Ni sites with more than one coordinating N, the spin moment is reduced to zero, and hence other effects may dominate their adsorption behaviors,^34^ resulting in difference in binding strength with intermediates. Notably, in all cases, the slope of Δ*E*_O*_ is about twice those of Δ*E*_HO*_ and Δ*E*_HOO*_, while the trends for the latter two are approximately parallel. These features align with the established scaling relations between these adsorption energies.^35,36^ Since all the three intermediates bind to the active site * through the oxygen-end, their adsorption energies are linearly interrelated. The slope of the scaling relation roughly depends on the number of bonds formed between the intermediate and the metal site during adsorption, typically yielding a slope of ∼2 between Δ*E*_O*_ and Δ*E*_HO*_/Δ*E*_HOO*_, and ∼1 between Δ*E*_HO*_ and Δ*E*_HOO*_. As shown in Fig. 2d, the following relationships hold for the SACs in this study: Δ*E*_O*_ = 2.0Δ*E*_HO*_ + 0.94 eV and Δ*E*_HOO*_ = 1.0Δ*E*_HO*_ + 3.01 eV. These equations are consistent with previous reports on two-dimensional graphene-based oxygen electrocatalysts.^37^

Based on the existence of above scaling relations, we derived state-of-the-art microkinetic modeling for ORR and OER by exhaustively considering the key elementary steps (modeling details and the employed key parameters can be found in ref. 29 and 38–40).^29,38–40^ Compared to the conventional “theoretical overpotential models”,^41^ these models lead to better agreement with the observable performance indicators of OER and ORR due to the consideration of more complex information such as the derived coverage and microkinetics.^29,38–40,42,43^ It should be noted that pH effects are not considered in the present model. Accurately incorporating pH influence would require the explicit inclusion of electric-field effects, which remains computationally prohibitive. Nevertheless, the methodology presented here is general and can, in principle, be extended to pH-dependent analyses once such effects are included.^44^ As a result, OER and ORR activities (defined by *U*_RHE_ @ *j*_0_ = 10 mA cm^−2^ and turnover frequency (TOF) @ *U*_RHE_ = 0.9 V, respectively; RHE: reversible hydrogen electrode) both exhibit a volcano-shaped dependence on the adsorption free energy of any of the three oxygenated species (*e.g*., HO*).^36,38,41,42^ These trends quantitatively express the *Sabatier* principle, which states that best catalysts are located at the top of volcano plots and should neither have too strong nor too weak binding strength to reaction intermediates. As demonstrated in Fig. 2e, the optimal Δ*G*_HO*_ for OER catalysts is approximately 1.1 eV, leading to a minimum 1.60 V_RHE_ @ *j*_0_ = 10 mA cm^−2^. On the left-leg of the volcano, strong oxygen binding (*e.g*., FeN_4_) makes the transition of O* to HOO* the rate-determining step (RDS), whereas on the right-leg, weak oxygen binding (*e.g*., NiN_4_) shifts the RDS to HO* deprotonation. For 4e^−^ ORR catalysis (Fig. 2f), the ideal Δ*G*_HO*_ is near 0.8 eV @ 0.9 V_RHE_. On the left-leg of the volcano (*i.e*., the strong binding branch), the reaction rate is retarded by the desorption of HO*, while on the right-leg (*i.e*., the weak binding branch), the formation of HOO* becomes the bottleneck.

**Fig. 3:**
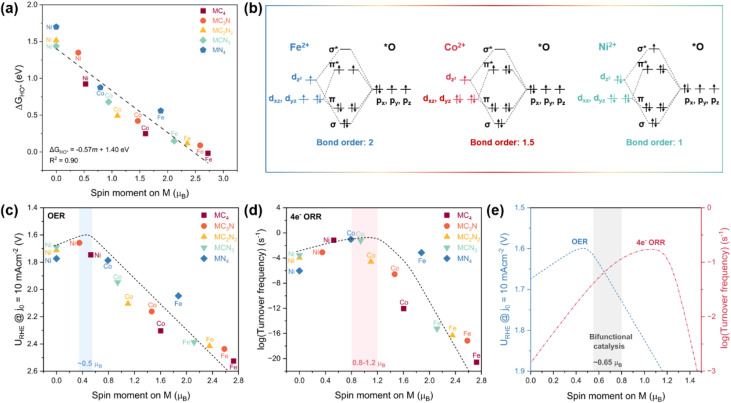
(a) Linear fit correlating HO* adsorption energy and spin moment (*m*) of Fe/Co/Ni sites in M–N–C SACs. The black dashed line and the formula Δ*G*_HO*_ = −0.57*m* + 1.40 eV are obtained from linear fitting of the data. (b) Molecular orbital diagrams illustrating the interactions between the adsorbed O and the IS Fe^2+^, LS Co^2+^, and LS Ni^2+^ sites. (c) OER and (d) ORR activities of M–N–C SACs as a function of spin moment on the metal site. The corresponding volcano models (black dashed lines in (c) and (d), respectively) are constructed based on the linear relationship shown in (a). Light blue and red shaded regions indicate the optimal spin moment ranges for OER and ORR, respectively. (e) Predicted trends in OER/ORR activity governed by the spin moment of Fe/Co/Ni sites. The gray shaded region represents the optimal spin-moment window for bifunctional oxygen electrocatalysis.

As shown in Fig. 3a and S5, the linear fitting results reveal that the coefficient of determination (*R*^2^) between the HO* adsorption energy and the spin magnetic moment of the metal site is 0.90, suggesting that the spin moment is a promising oxygen electrocatalysis descriptor for sing-atom Fe/Co/Ni–N–C catalysts. Indeed, volcano-shaped curves were also plotted between OER/ORR activities and the metal-site spin moment, peaking at ∼0.5 *µ*_B_ for OER and ∼1.0 *µ*_B_ for ORR, respectively (Fig. 3c and d). This finding is analogous to the e_g_-filling descriptor identified for transition metal oxides, where octahedral symmetry renders the d orbitals splitting into a lower-energy triplet (t_2g_) and a higher-energy doublet (e_g_).^2,3,45^ In that scenario, the electron occupancy in the e_g_ orbitals (d_*z*_^2^ and d_*x*_^2^_−*y*_^2^) predominantly interacting with O 2p orbitals governs the binding strengths with oxygen-related adsorbates, with an e_g_ filling near unity yielding the peak activity. In the case of two-dimensional M–N–C materials, the planar square symmetry leads to a different d-orbital splitting pattern (Fig. 1d), where the d_*z*_^2^, d_*xz*_, and d_*yz*_ orbitals are identified as the frontier orbitals hybridizing with the 2p orbitals of the adsorbed oxygen atom during chemisorption.^23,46,47^ The spin moment of the metal center provides a direct and quantifiable measure of the unpaired electron population within these frontier orbitals. A lower spin moment, reflecting fewer unpaired electrons, systematically increases the occupancy of antibonding states formed upon metal–oxygen orbital interaction (Fig. 3b).^7,23,25,48^ According to bond order theory, where bond order is defined as half the difference between the number of bonding and antibonding electrons,^7^ this increase in antibonding occupancy leads to a reduced bond order, indicating weaker adsorption. This theoretical framework underpins the linear correlations observed between the adsorption energies of oxygenate intermediates and the spin moments of M–N–C SACs (Fig. 3a and S5). Consequently, an intermediate spin moment corresponds to moderate adsorption strength, which optimizes catalytic activity in accordance with the Sabatier principle (Fig. 3c and d).

Beyond regulating adsorption/desorption thermodynamics through orbital interactions, the spin configurations of active centers also influence the electron transfer between the active sites and the reaction intermediates.^7,18^ This spin-dependent behavior arises because the oxygen molecule has a triplet ground state (↑O=O↑) with two unpaired electrons, whereas its evolution from or reduction to diamagnetic water/hydroxide involves spin flipping and therefore requires spin-related electron transfer.^7,18^ Specifically, spin-polarized electron accumulation at the catalyst surface can establish spin-selective transport channels. These channels facilitate the transfer of electrons with the appropriate spin orientation, thereby enhancing charge mobility during oxygen electrocatalysis. Consequently, ferromagnetic materials with metal centers at higher spin states are more likely to support the formation of such spin-polarized channels, promoting more efficient electron transport and thus improving overall reaction kinetics.^6,7^

Therefore, the overall catalytic activity is impacted by two distinct spin-related effects, which both can be correlated with the magnetic moment yet impose different requirements on spin characteristics.^7,18^ On one hand, spin-state regulation optimizes the adsorption strengths of intermediates *via* orbital interactions, favoring moderate spin moments to achieve optimal binding energies. On the other hand, spin polarization enhances selective electron transport, which benefits from larger spin moments by broadening spin-related channels and improving charge transfer efficiency. The relative dominance of these contributions is inherently catalyst-dependent.

For M–N–C SACs, the absence of long-range magnetic ordering limits their intrinsic spin polarization compared to bulk ferromagnetic materials, constraining the establishment of effective spin-selective electron transfer channels. However, this limitation can be offset by the high electrical conductivity of the carbon-rich matrix, which ensures efficient charge transport throughout the catalyst architecture. As a result, the reaction kinetics in such systems are more strongly governed by thermodynamic descriptors (such as the adsorption free energy of intermediates), where a lower magnetic moment can lead to enhanced catalytic activity, provided that the thermodynamic benefit of weakened HO* adsorption outweighs the marginal reduction in spin-related channel width.^7,18^

According to the linear correlation between Δ*G*_HO*_ and spin moment shown in Fig. 3a, Δ*G*_HO*_ can be predicted with a given spin moment. Then based on the linear correlations among Δ*G*_HO*_, Δ*G*_O*_, and Δ*G*_HOO*_, the latter two also can be derived from the value of spin moment. With the adsorption energies of all the three intermediates known, the corresponding theoretical activities of oxygen electrocatalysis can be predicted. Interestingly, as shown in Fig. 3c and d, the optimal spin moments for OER/ORR are 0.5 and 1.0 *µ*_B_, respectively. For bifunctional catalysis, the optimal spin moment is around 0.65 *µ*_B_, which corresponds to a commendable reactivity for both OER and ORR (Fig. 3e).

Consequently, the OER/ORR activities of single-atom Fe/Co/Ni sites can be optimized by tailoring the spin moment. For instance, N doping lowers the spin moment of FeC_4_ site, weakening the excessively strong HO* binding and thereby enhancing the catalytic activity. To further enhance the OER/ORR performance of single-atom Fe active center, the spin moment of Fe should be tuned below that of FeN_4_ (1.88 *µ*_B_). This inference is consistent with experimental reports that reducing the on-site magnetic moment of FeN_4_ site *via* enriching graphitic N can bring about higher intrinsic catalytic activity for ORR.^25^ Conversely, for Ni–N–C SACs that bind oxygen-related intermediates too weakly, moderately increasing the spin moment above that of NiC_4_ (0.52 *µ*_B_) would improve the ORR performance. Moreover, SACs with spin moments between 0.5 and 0.8 *µ*_B_ (*e.g*., CoN_4_ and NiC_4_) are the candidates for bifunctional oxygen electrocatalysis, which simultaneously exhibit decent performance for both OER and ORR.

While a full pH-dependent analysis is computationally demanding, the current pH-independent model serves its purpose as a necessary and valid first-order approximation to establish the proof-of-concept for the spin-moment descriptor. This approach is justified because the fundamental physics governing the correlation between spin states and adsorption energetics, specifically the d-orbital occupancy and its influence on metal–oxygen bonding, is inherently pH-independent. Although the exact numerical values of the optimal spin moments may exhibit minor shifts when solvation and field effects are fully accounted for,^39,44^ the existence of an optimal spin window and the monotonic relationship between spin moment and adsorption strength remain unchanged. Therefore, the fundamental design principle of tuning the spin moment to achieve optimal adsorption strength is expected to hold across a wide range of pH conditions. Future refinements will focus on benchmarking these optimal values under experimentally relevant pH environments.

### 2.2 Spin moment descriptor-guided screening of dual-atom catalysts for bifunctional oxygen electrocatalysis

**Fig. 4:**
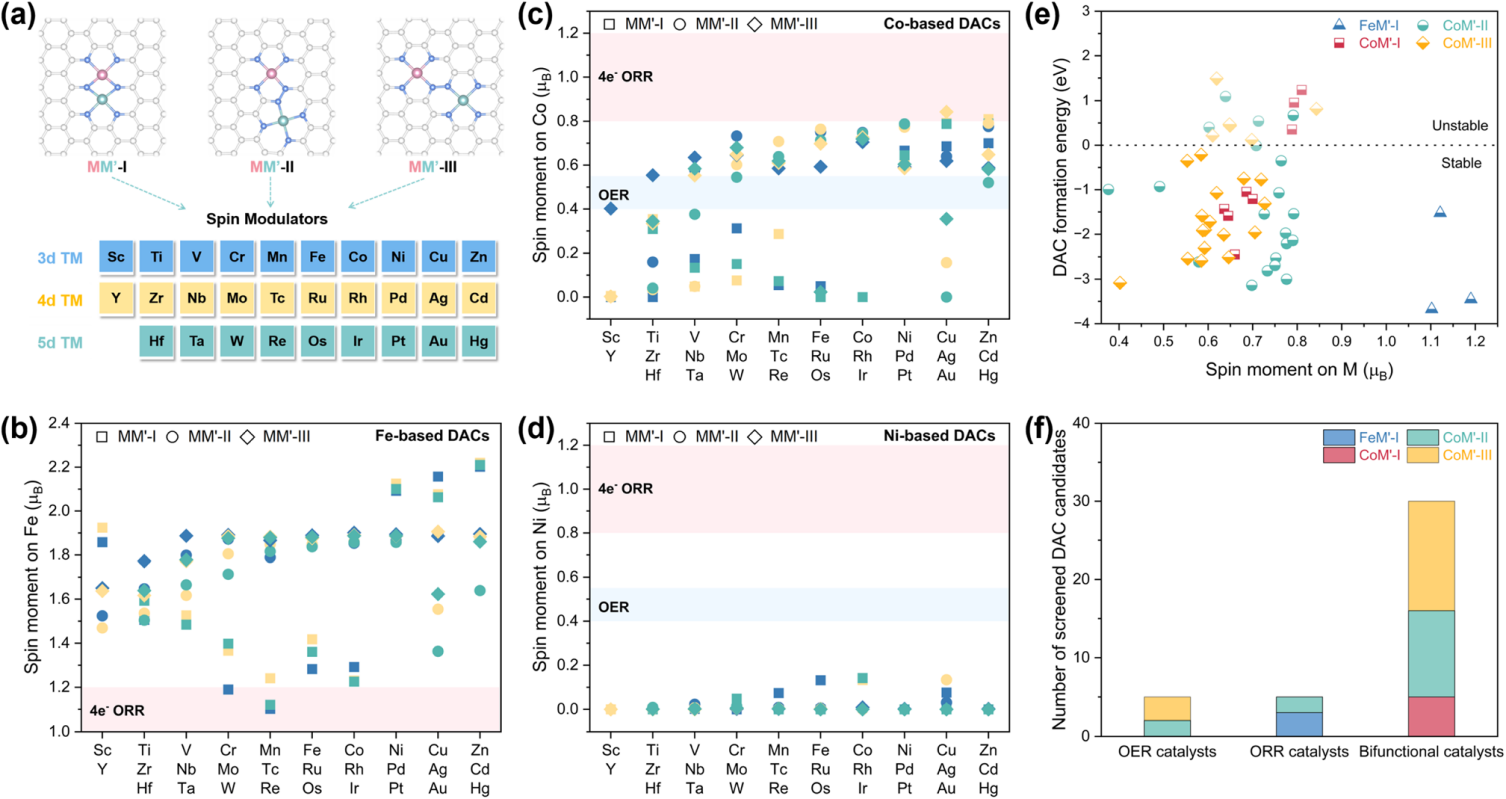
(a) Spin engineering of M (M = Fe/Co/Ni) atoms *via* introducing additional TM atom M′ in the second, third, and fourth coordination shell, respectively. N, M, and M′ atoms are colored blue, pink, and turquoise, respectively. Magnetic moments of (b) Fe, (c) Co, and (d) Ni atoms in MM′-I, MM′-II, and MM′-III DAC frameworks. The M′ spin modulators (3d, 4d, and 5d TMs) are colored blue, yellow, and turquoise, respectively. The MM′-I, MM′-II, and MM′-III DACs are represented by square, circle, and diamond-shaped symbols, respectively. The pink and blue shaded areas in (b–d) indicate target spin moment regions for ORR and OER catalyst screening, respectively. (e) Formation energies of MM′-I, MM′-II, and MM′-III DACs with M sites at optimal spin states for oxygen electrocatalysis. The light-colored symbols are thermodynamically unstable. (f) Number of screened DAC candidates for OER, ORR, and bifunctional oxygen electrocatalysis.

In practice, a nitrogen-rich first-shell coordination environment is typically indispensable for anchoring single-atom metal sites. To further enhance the OER/ORR catalytic activity and nuancedly modulate the spin moment of Fe/Co/Ni (M) sites, we introduced additional modulation atoms in the form of a second transition metal (M′) located in the vicinity of the MN_4_ site. As shown in Fig. 4a, TMs from the 3d, 4d, and 5d periods were employed as spin modulators to regulate the spin states of central M atom *via* electronic interaction effects.^21,27,49,50^ Besides the identity of the introduced metal atom, its spatial arrangement around the central M site also can alter the spin modulation effects.^27,51^ To prevent the direct participation of M′ in oxygen catalysis as reactive sites, the introduced M′ atom is placed in the second and the third coordination shell of M to construct MM′-I, MM′-II, and MM′-III dual-metal atomic models, respectively. In the first configuration, M and M′ atoms are bridged by two N atoms, yielding a M–M′ interatomic distance (*d*_MM′_) of ∼2.3 Å. In the latter two structures, M and M′ atoms are atomically dispersed, with a d_MM′_ of ∼4.5 and ∼5.0 Å, respectively.

As shown in Fig. 4b, the introduction of an adjacent TM in the MM′-I framework (square symbols) successfully reduces the spin moment on Fe. Notably, Cr, Mn, and Re are particularly effective in lowering the spin moment to ∼1.1 *µ*_B_, which favors ORR activity improvement yet remains undesirable for OER catalysis. In contrast, Co-based DACs exhibit great potential for both OER and ORR, with the spin moments of Co sites distributed between 0 and 0.8 *µ*_B_ (Fig. 4c). This is reasonable as the CoN_4_ site in SAC counterpart has a spin moment of 0.79 *µ*_B_, a value that lies within the optimal range for oxygen electrocatalysis, resulting in the fine-tuning within DAC frameworks more feasible. In the case of Ni-based DACs, the multiple coordination engineering strategies illustrated in Fig. 4a fail to significantly raise the spin moment of NiN_4_ site above 0.2 *µ*_B_. Alternative approaches, such as strain engineering and axial coordination regulation, may be helpful to enhance the spin moment of Ni sites.^20,30–32^

Guided by the target magnetic moments (specifically, 0.4–0.55 *µ*_B_ for OER, 0.8–1.2 *µ*_B_ for ORR, and 0.55–0.8 *µ*_B_ for bifunctional oxygen electrocatalysis), DACs with high potential for efficient catalysis were identified. Additionally, the thermodynamic feasibility of these candidates further assessed by calculating their formation energies (summarized in Tables S2–S10). A more negative formation energy corresponds to a more thermodynamically stable DAC configuration. Ultimately, 40 DACs exhibiting both negative formation energy and optimized spin moments were identified as promising candidates (Fig. 4e). Among these, five are targeted for the OER, five for the ORR, and 30 for bifunctional oxygen electrocatalysis (Fig. 4f).

**Fig. 5:**
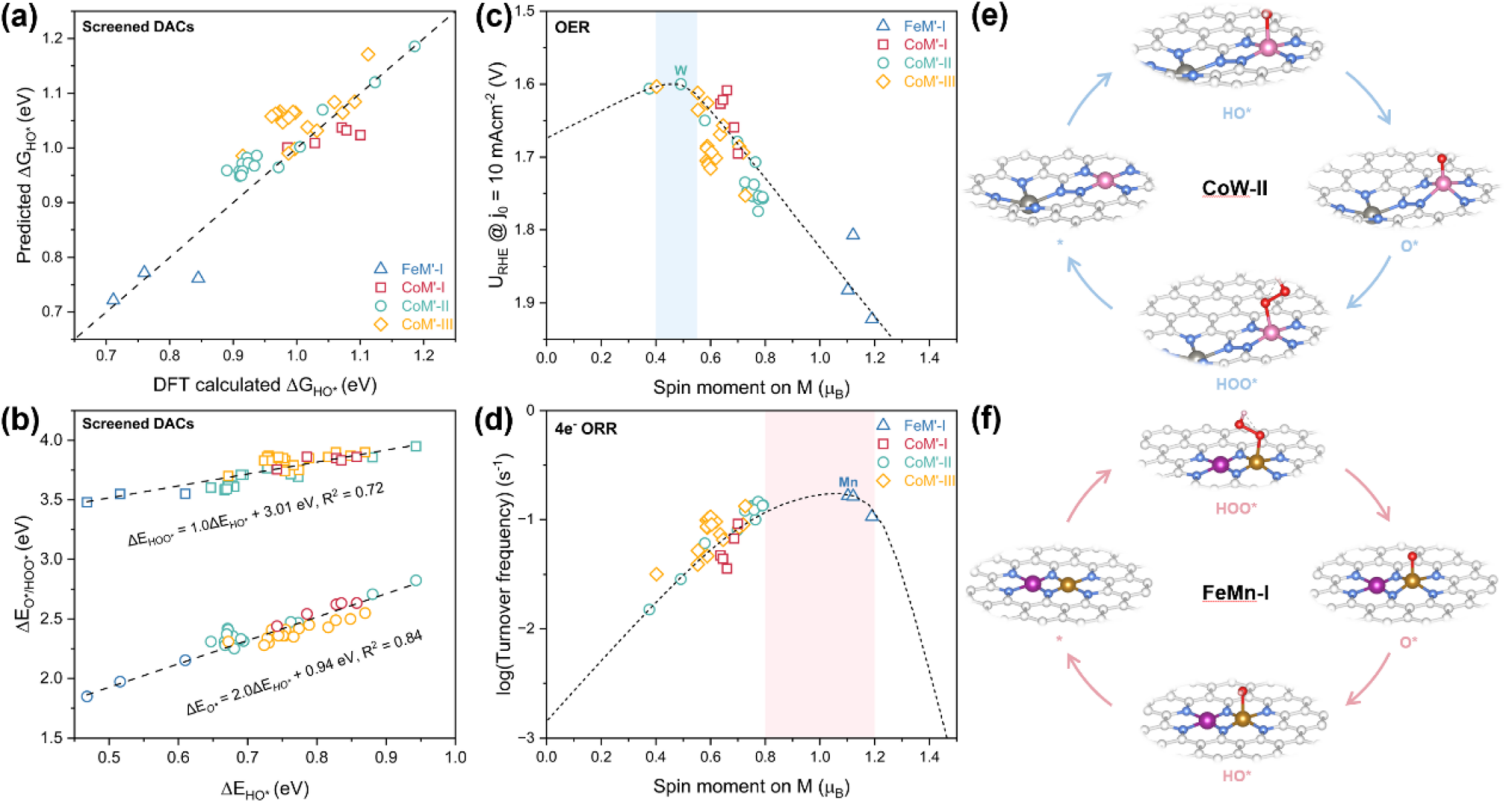
(a) Spin-moment-predicted and DFT-calculated Δ*G*_HO*_ of single-atom M sites within the screened DACs. Diagonal dashed line is included as visual guide. (b) Scaling relations between DFT-calculated Δ*E*_O*_/Δ*E*_HOO*_ and Δ*E*_HO*_ of the screened DACs. The dashed black lines and equations are obtained from linear fitting of the data. (c) OER and (d) ORR activity of single-atom M sites within the screened DACs as a function of spin moment on Fe/Co/Ni site. Illustration of the reaction steps on the optimal screened DACs (CoW-II and FeMn-I) for (e) OER and (f) ORR, respectively.

We further calculated the energies of oxygenate intermediates adsorbed exclusively on the M sites in these screened DACs and obtained the corresponding DFT-calculated results (summarized in Tables S11–S13). As shown in Fig. 5a, for most screened DACs, the HO* adsorption energy obtained from DFT calculations agree well with those predicted using the spin moment descriptor. This consistency demonstrates the reasonable predictive capability of the spin moment descriptor for rapidly screening single-atom active sites within the three types of DAC configurations. Some deviations between the two sets of performance values were observed, which may be attributed to the following factors. First, the spin moment descriptor makes predictions based on a linear scaling slope of −0.57 between Δ*G*_HO*_ and the spin moment. While this relation holds well for Fe-based systems, it is relatively less precise for Co- and Ni-based systems (Fig. 2a–c). Second, the introduced TM may indirectly influence the adsorption behavior of oxygen-related species on the central M site *via* long-range electronic interactions, thereby modifying the adsorption energy of intermediates. Third, in addition to electronic effects, the introduction of a neighboring TM atom can induce a strain effect due to the mismatch between the atomic radius of the dopant and the size of the vacancy in the graphene substrate, which may also contribute to differences in Δ*G*_HO*_.

In addition, as the scaling relations of the screened DACs are in agreement with those of SACs (Fig. 5b and 2d), the adsorption of intermediates on M sites with DAC configurations obey the “standard” single-site adsorption behavior. We add the points of screened DACs to the OER/ORR volcano models. As shown in Fig. 5c, CoW-II stands out for the OER, exhibiting 1.60 V_RHE_ @ *j*_0_ = 10 mA cm^−2^, significantly outperforming the CoN_4_ SAC site (1.79 V_RHE_ @ *j*_0_ = 10 mA cm^−2^). This improvement is linked to a reduction in the spin moment on the Co site from 0.79 *µ*_B_ in the SAC to ∼0.5 *µ*_B_ in the CoW-II DAC configuration. The decreased spin moment optimizing the oxygen binding strength, thereby facilitating the formation of HOO*. For the ORR, FeMn-I emerges as the most active catalyst. Compared to the FeN_4_ SAC site, the spin moment on the Fe site in FeMn-I is reduced to approximately 1.1 *µ*_B_, remarkably weakening the adsorption of oxygenate intermediates. Consequently, the 4e^−^ ORR activity is significantly improved. Furthermore, 16 Co-based DACs, with configuration I (M′ = Ni, Cu, Zn, Pd and Pt), II (M′ = V and Mn), and III (M′ = V, Cr, Co, Cu, Zn, Pd, Ta, Ir, and Pt), are identified as promising bifunctional oxygen catalysts (Table S13). These candidates, spanning three configurations, demonstrate good activity for both OER (with *U*_RHE_ ≤ 1.70 V @ *j*_0_ = 10 mA cm^−2^) and ORR.

## 3 Conclusions

In summary, we have systematically modulated the spin states of a series of single-atom Fe/Co/Ni active sites and investigated the influence of spin moments on their OER/ORR catalytic activities. Through varying the coordinating N atoms in the first coordination shell of Fe/Co/Ni center, a monotonic enhancement in the adsorption strengths of oxygenated intermediates on the metal site with increasing spin magnetic moment was revealed. Accordingly, a volcano-type correlation was established between the OER/ORR activity and the spin moment, peaking near 0.5 *µ*_B_ for OER and 1.0 *µ*_B_ for 4e^−^ ORR, respectively. With the introduction of additional TM atom in the second coordination shell, a finer control over the spin moment on the Fe/Co/Ni center was achieved. Guided by optimal spin moment values and considerations of synthesis feasibility, the screening process quickly yielded five DACs as promising OER catalysts and five as promising ORR catalysts. Among them, CoW-II for OER and FeMn-I for 4e^−^ ORR catalysis stood out. Furthermore, metal sites with spin moments ∼0.65 *µ*_B_ exhibited commendable performance for both OER and ORR, making them attractive as bifunctional oxygen electrocatalysts. These findings provide important insights into spin engineering of single magnetic atoms *via* regulating local coordination environment, and offer guidance for future rational design of highly active Fe/Co/Ni sing-atom catalysts in oxygen-related electrocatalytic applications.

## 4 Computational details

Spin-polarized density functional theory (DFT) calculations were performed using the Vienna *ab initio* simulation package (VASP).^52,53^ The projector augmented wave (PAW) method was employed to describe the interaction between ions and electrons, while the generalized gradient approximation (GGA) in the form of the revised Perdew–Burke–Ernzerhof (RPBE) functional was adopted for electron exchange-correlation.^54,55^ A plane-wave cutoff energy of 450 eV was used for both static calculations and structural optimizations. To model M–N–C (M = Fe/Co/Ni) SACs, a pristine graphene layer consisting of sixty carbon atoms was constructed. Two adjacent carbon atoms were removed to anchor a metal atom M, and *x* (*x* = 0–4) carbon atoms in the first coordination shell of M were substituted with nitrogen atoms. For DAC models, four carbon atoms were removed from pristine graphene to host two adjacent transition metal atoms, and the four nearest carbon atoms surrounding each metal site were replaced with nitrogen atoms. All structures were separated by a vacuum layer of 20 Å along the non-periodic direction to avoid spurious interactions between periodic images. The van der Waals interaction of adsorbates was described by the zero damping DFT-D3 method of Grimme.^56^ The Brillouin zone was sampled using Monkhorst–Pack *k*-point grids: a gamma-centered 2 × 2 × 1 mesh for geometry relaxation and a 5 × 5 × 1 mesh for electronic structure calculations, respectively. Convergence criteria were set to 0.05 eV Å^−1^ for atomic force and 10^−5^ eV for total energy.

The electric binding energies of the reaction intermediates (HO*, O*, and HOO*) in conventional 4e^−^ OER/ORR mechanisms are calculated by the following equations:Δ*E*_HO*_ = *E*_HO*_ − E_*_ − (*E*_H_2_O_ − 1/2*E*_H_2__)Δ*E*_O*_ = *E*_O*_ − E_*_ − (*E*_H_2_O_ − *E*_H_2__)Δ*E*_HOO*_ = *E*_HOO*_ − E_*_ − (2*E*_H_2_O_ − 3/2*E*_H_2__)where E_*_, *E*_HO*_, *E*_O*_, and *E*_HOO*_ are energies of the pristine catalyst surface, and surface adsorbed by HO*, O*, and HOO* species, respectively. *E*_H_2_O_ and *E*_H_2__ represent the energies of a H_2_O and a H_2_ molecule in vacuum, respectively.

The binding free energies of these adsorbates are determined using the computational hydrogen electrode (CHE) method,^41^ with the following equationΔ*G*_ads_ = Δ*E*_ads_ + ΔZPE − *T* × Δ*S* + *E*_solv_where Δ*E*_ads_, ΔZPE, Δ*S*, and *E*_solv_ are the binding energy of the adsorbate, the difference in zero-point energies, the variation in entropy during the reaction, and solvation corrections, respectively.^29^*T* is the temperature (298.15 K).

The microkinetic modeling of the ORR and OER models was conducted based on the methodology described by Zhang *et al.*,^29,38,39^ Hansen *et al.*,^57^ Kelly *et al.*,^43^ and Dickens *et al.*,^40^ using our self-developed codes. These models are also deployed in our Digital Catalysis Platform (*DigCat*: http://www.digcat.org/).^58^ The energies used in the microkinetic modeling were adjusted using the scaling relations presented in Fig. 2d in the main text. Our kinetic model implicitly accounts for reaction energy barriers through Brønsted–Evans–Polanyi (BEP) relationships.^59^ We note that explicit calculation of activation barriers, which are important for activity assessment and influenced by factors such as solvent reorganization energy and the effective overlap between electrode and reactant orbitals,^60,61^ lies beyond the scope of the present study and will be addressed in future work.

The d band center gap (Δ*d*) of spin-polarized M–N–C (M = Fe/Co/Ni) SACs is defined as
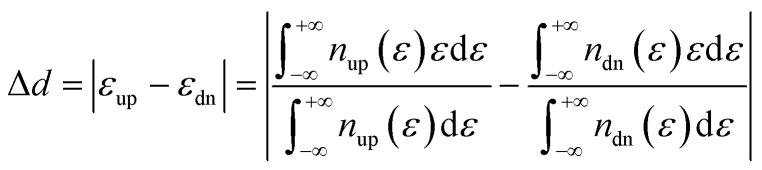
where *ε*_up_ and *ε*_up_ are the d-band center of the M 3d spin-up and spin-down pDOSs, respectively.^23^

The formation energy (*E*_f_) of DACs incorporating two transition metal atoms (denoted as M and M′, respectively) is computed using the following equation:*E*_f_ = *E*_DAC_ − *E*_M@sub_ − *E*_M′-bulk_where *E*_DAC_ represents the total electronic energy of the DAC system, *E*_M@sub_ corresponds to the energy of the substrate containing a single M (M = Fe/Co/Ni) atom as the active site and an adjacent vacancy for further metal incorporation, and *E*_M′-bulk_ is the energy of single M′ atom in its most stable bulk phase. This formulation quantifies the energy change associated with introducing the spin modulator M′ atom into the M-embedded substrate to form the DAC, thereby providing a measure of the stability of the dual-atom configuration.

## Conflicts of interest

There are no conflicts to declare.

## Supplementary Material

SC-017-D6SC00439C-s001

## Data Availability

All data are available in the supplementary information (SI) and the Digital Catalysis Platform (DigCat, http://www.digcat.org/). Supplementary information is available. See DOI: https://doi.org/10.1039/d6sc00439c.
