# Supplementary material for: Spin effect regulation as a design principle for M–N–C catalysts for oxygen electrocatalysis
Source: Chem Sci. 2026 Mar 30;17(16):7947–57. doi: 10.1039/d6sc00439c (PMC13054587; doi:10.1039/d6sc00439c)
Supplement: SC-017-D6SC00439C-s001 [file SC-017-D6SC00439C-s001.pdf]

Supplementary Information (SI) for Chemical Science.
This journal is © The Royal Society of Chemistry 2026

Supporting Information

Spin Effect Regulation as A Design Principle for M-N-C Catalysts for Oxygen Electrocatalysis

Haiyan Li[abc], Zhanzhao Fu[a], Yuan Yuan[d], Di Zhang[e], Yubo Chen[cf], Hao Li[*e], and Ang Cao[*ag]

[a] State Key Laboratory of Clean Energy Utilization, College of Energy Engineering, Zhejiang University, Hangzhou 310027, China

[b] Key Laboratory of Biomass Chemical Engineering of Ministry of Education, College of Chemical and Biological Engineering, Zhejiang University, Hangzhou 310027, China

[c] Hydrogen Energy Institute, Zhejiang University, Hangzhou 310027, China

[d] Suzhou MatSource Technology Co., Ltd., Suzhou 215000, China

[e] Advanced Institute for Materials Research (WPI-AIMR), Tohoku University, Sendai 980-8577, Japan

[f] Institute of Advanced Equipment, College of Energy Engineering, Zhejiang University, Hangzhou 310027, China

[g] Inner Mongolia Daqingshan Laboratory, Hohhot 017000, China

*E-mail: li.hao.b8@tohoku.ac.jp (H.L.); angc@zju.edu.cn (C.A.)

Microkinetic Modeling of ORR/OER on M-N-C Catalysts

The microkinetic modeling of the ORR and OER models was conducted based on the methodology described by Zhang et al.,[1] Hansen et al.,[2] Kelly et al.,[3] and Dickens et al.,[4] using our self-developed codes. These models are also deployed in our Digital Catalysis Platform (*DigCat*: www.digcat.org).[5] Rates for intermediate steps were calculated using Equation (1):

$$rate = k_f \prod \theta_{reac} - k_r \prod \theta_{prod}$$ MERGEFORMAT

(1)

where θ_{reac} and θ_{prod} are the coverages of reactants and products, respectively. The rate constant *k* was calculated as the function of reaction perfector A (s^{-1}), activation free energy G_a, Boltzmann constant k_B, and reaction temperature *T*:

$$k = Ae^{-\frac{G_a}{k_B T}}$$ MERGEFORMAT

(2)

The intermediate reactions considered in the modeling of the $4e^-$ ORR volcano are shown in Reactions (3)-(8):

$$O_2(aq) \rightarrow O_2(dl)$$ MERGEFORMAT

(3)

$$O_2(dl) + * \rightarrow O_2*$$ MERGEFORMAT

(4)

$$O_2* + H^+ + e^- \rightarrow HOO*$$ * MERGEFORMAT (5)

$$HOO* + H^+ + e^- \rightarrow O* + H_2O(l)$$ MERGEFORMAT

(6)

$$O* + H^+ + e^- \rightarrow HO*$$ MERGEFORMAT

(7)

$$HO* + H^+ + e^- \rightarrow H_2O(l) + *$$ MERGEFORMAT

(8)

Our ORR volcano plot is based on the mechanism where the proton source is considered to be water molecules, even under alkaline conditions, rather than hydroxide ions. We believe this mechanism is supported by extensive experimental validation, including

studies on transition metals[3, 6, 7], transition metal oxides[8], and single atoms[1, 9, 10].

The intermediate reactions considered in the microkinetic modeling of the OER volcano are shown in Reactions (9)-(14):

$$H_2O(l) + * \rightarrow H_2O* \tag{9}$$

$$H_2O* \rightarrow HO* + H^+ + e^- \tag{10}$$

$$HO* \rightarrow O* + H^+ + e^- \tag{11}$$

$$O* + H_2O(l) \rightarrow HOO* + H^+ + e^- \tag{12}$$

$$HOO* \rightarrow O_2* + H^+ + e^- \tag{13}$$

$$O_2* \rightarrow * + O_2(g) \tag{14}$$

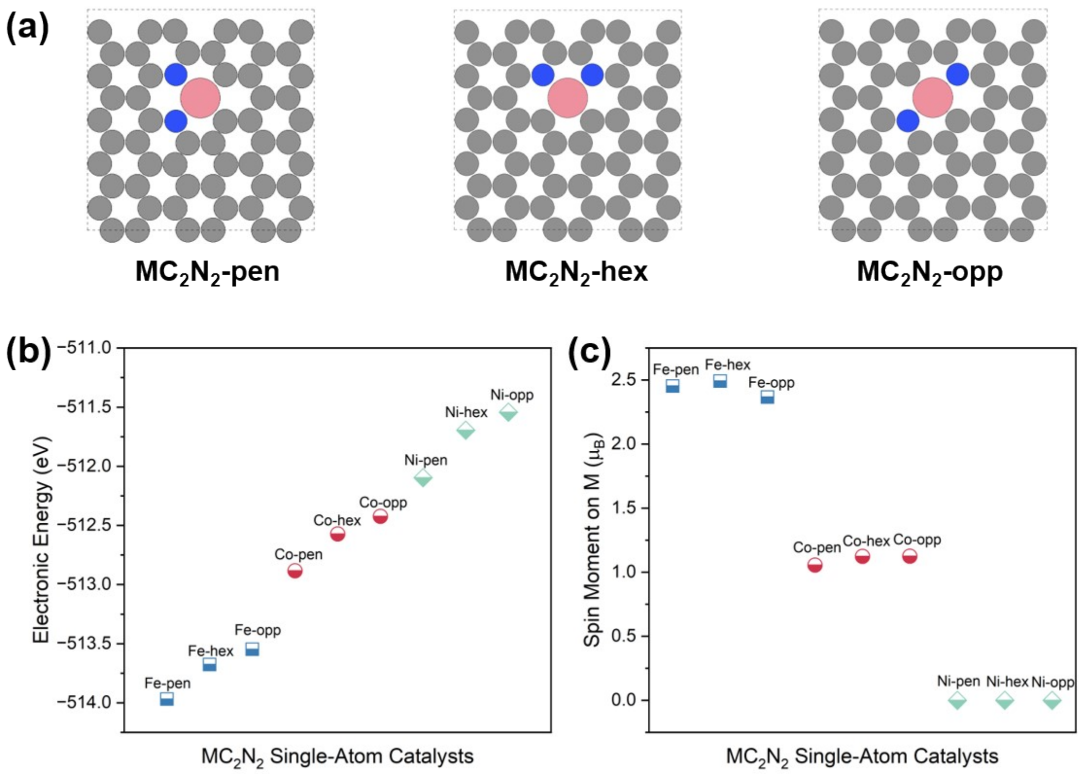

Figure S1. (a) Atomic models, (b) electronic energy, and (c) metal spin moment of MC_2N_2 (M = Fe/Co/Ni) with different configurations. As demonstrated in Figure S1c, the specific arrangement of the two coordinated N dopants has a negligible influence on the spin moment of the central metal atom. Additionally, for either Fe, Co, or Ni, the MC_2N_2-pen configuration possesses the lowest energy among the three structural models considered. Therefore, the MC_2N_2-pen configuration is selected for further investigation.

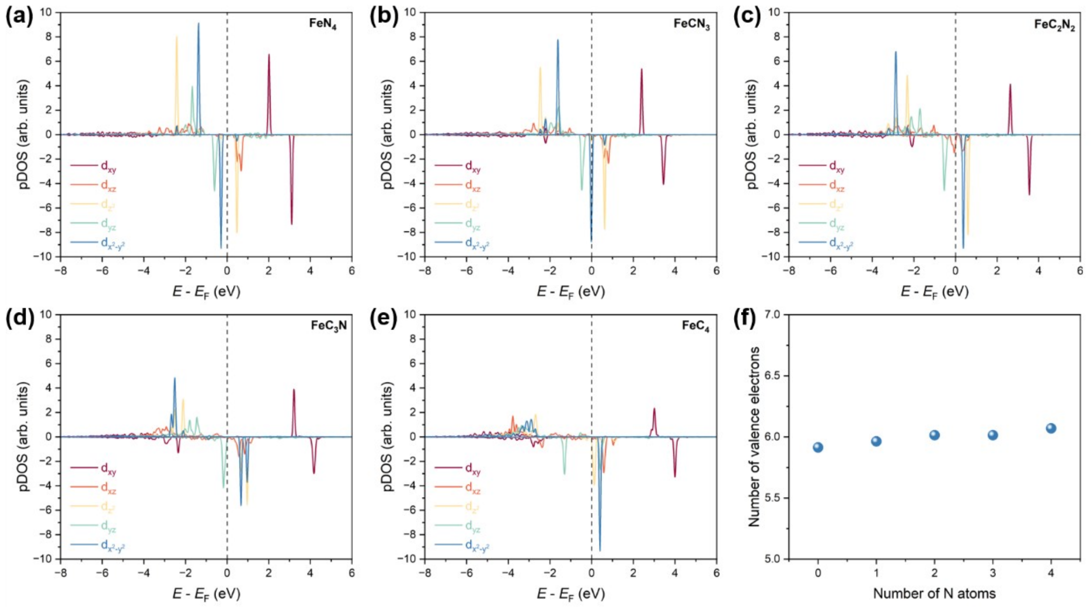

Figure S2. (a-e) Fe 3d orbital pDOS plots for Fe-N-C SACs. (f) The corresponding number of valence electrons on the Ni atom as a function of its nitrogen coordination number.

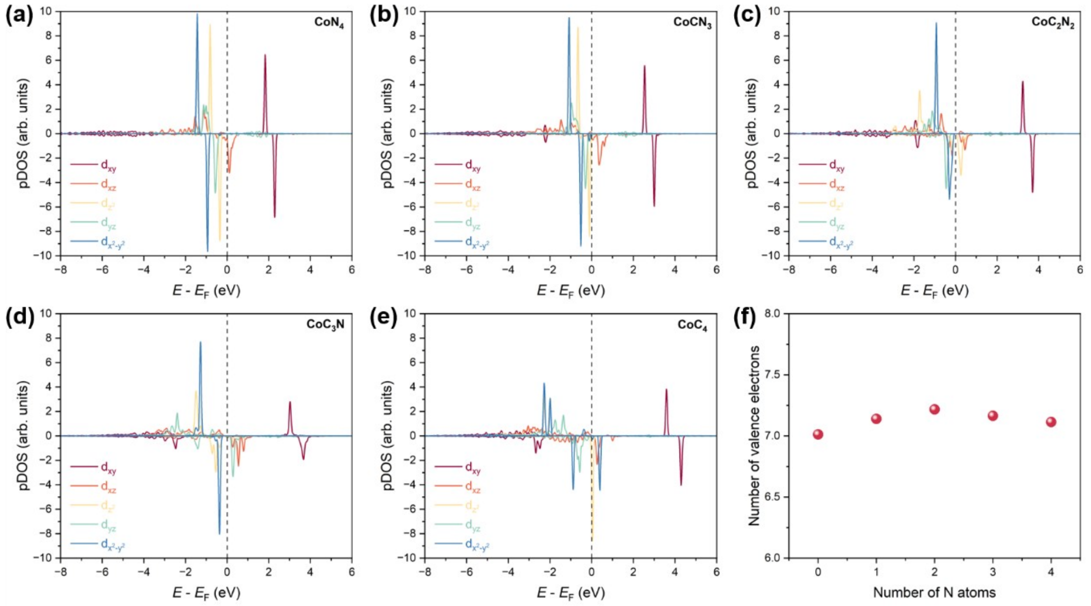

Figure S3. (a-e) Co 3d orbital pDOS plots for Co-N-C SACs. (f) The corresponding number of valence electrons on the Ni atom as a function of its nitrogen coordination number.

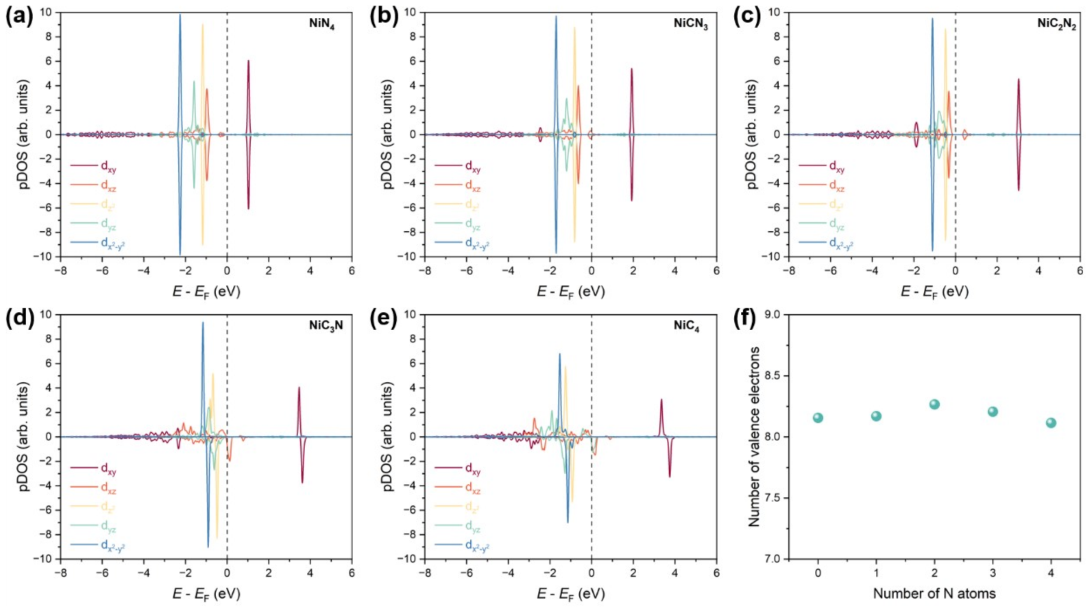

Figure S4. (a-e) Ni 3d orbital pDOS plots for Ni-N-C SACs. (f) The corresponding number of valence electrons on the Ni atom as a function of its nitrogen coordination number.

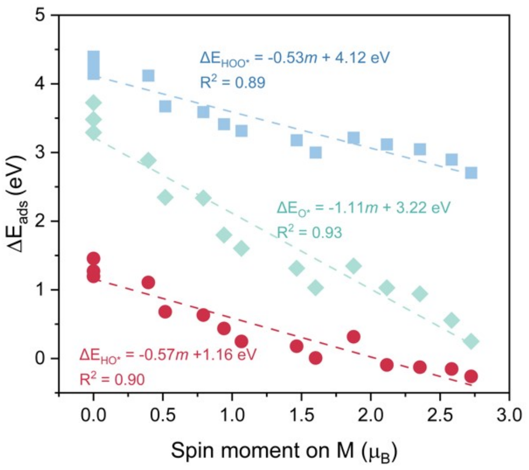

Figure S5. HO*/O*/HOO* binding energies as a function of the metal spin moment of M-N-C SACs.

Table S1. Spin moments, DFT-calculated oxygenate intermediate binding energies, and ORR/OER activities of M-N-C SACs.

SACs	Spin moment (μB)	ΔE_{HO*} (eV)	ΔE_{O*} (eV)	ΔE_{HOO*} (eV)	TOF @ 0.9 V_{RHE} (s^{-1})	U_{RHE} @ 10 $mAcm^{-2}$ (V)
FeC_4	2.72	-0.26	0.25	2.70	-19.778	2.525
FeC_3N	2.58	-0.15	0.56	2.90	-16.354	2.437
FeC_2N_2	2.35	-0.13	0.94	3.05	-15.52	2.415
$FeCN_3$	2.12	-0.09	1.03	3.12	-14.437	2.387
FeN_4	1.88	0.32	1.35	3.22	-3.092	2.047
CoC_4	1.60	0.01	1.03	3.00	-11.243	2.304
CoC_3N	1.47	0.18	1.31	3.18	-5.911	2.162
CoC_2N_2	1.07	0.25	1.60	3.32	-4.318	2.105
$CoCN_3$	0.94	0.44	1.80	3.41	-1.248	1.948
CoN_4	0.79	0.63	2.34	3.59	-0.816	1.786
NiC_4	0.52	0.68	2.35	3.67	-0.894	1.745
NiC_3N	0.40	1.11	2.89	4.12	-2.605	1.658
NiC_2N_2	0.00	1.27	3.29	4.23	-3.497	1.712
$NiCN_3$	0.00	1.20	3.48	4.15	-3.053	1.687
NiN_4	0.00	1.46	3.73	4.40	-5.853	1.773

Table S2. Spin moments and formation energies of FeM'-I DACs. The screening results for OER/ORR catalysts are highlighted.

3d TM	Fe spin moment (μB)	Formation Energy (eV)	4d TM	Fe spin moment (μB)	Formation Energy (eV)	5d TM	Fe spin moment (μB)	Formation Energy (eV)
Sc	1.86	-4.28	Y	1.92	-4.01			
Ti	1.51	-4.34	Zr	1.60	-3.91	Hf	1.59	-3.74
V	1.49	-3.81	Nb	1.53	-2.96	Ta	1.48	-2.73

**Cr**	**1.20**	**-3.45**	Mo	1.37	-2.01	W	1.40	-3.78
**Mn**	**1.10**	**-3.68**	Tc	1.24	-2.04	**Re**	**1.12**	**-1.52**
Fe	1.28	-2.88	Ru	1.42	-1.96	Os	1.36	-1.47
Co	1.29	-2.61	Rh	1.23	-2.11	Ir	1.23	-1.74
Ni	2.09	-2.20	Pd	2.13	-1.20	Pt	2.10	-1.28
Cu	2.16	-0.85	Ag	2.08	1.22	Au	2.06	0.63
Zn	2.20	-0.99	Cd	2.22	1.78	Hg	2.21	5.09

Table S3. Spin moments and formation energies of CoM'-I DACs. The screening results for OER/ORR catalysts are highlighted.

**3d TM**	**Co spin moment (μB)**	**Formation Energy (eV)**	**4d TM**	**Co spin moment (μB)**	**Formation Energy (eV)**	**5d TM**	**Co spin moment (μB)**	**Formation Energy (eV)**
Sc	0.00	-4.28	Y	0.10	-4.01			
Ti	0.00	-4.34	Zr	0.29	-3.91	Hf	0.03	-3.74
V	0.17	-3.81	Nb	0.05	-2.96	Ta	0.04	-2.73
Cr	0.31	-3.45	Mo	0.08	-2.01	W	0.15	-3.78
Mn	0.05	-3.68	Tc	0.28	-2.04	Re	0.07	-1.52
Fe	0.05	-2.88	Ru	0.00	-1.96	Os	0.00	-1.47
Co	0.00	-2.61	Rh	0.00	-2.11	Ir	0.00	-1.74
**Ni**	**0.67**	**-2.20**	**Pd**	**0.64**	**-1.20**	**Pt**	**0.65**	**-1.28**
**Cu**	**0.69**	**-0.85**	Ag	0.79	1.22	Au	0.79	0.63
**Zn**	**0.70**	**-0.99**	Cd	0.81	1.78	Hg	0.79	5.09

Table S4. Spin moments and formation energies of NiM'-I DACs.

**3d TM**	**Ni spin moment (μB)**	**Formation Energy (eV)**	**4d TM**	**Ni spin moment (μB)**	**Formation Energy (eV)**	**5d TM**	**Ni spin moment (μB)**	**Formation Energy (eV)**
Sc	0.00	-5.15	Y	0.00	-4.82			

Ti	0.00	-4.77	Zr	0.00	-4.41	Hf	0.00	-4.26
V	0.00	-4.20	Nb	0.01	-3.25	Ta	0.01	-3.18
Cr	0.00	-3.80	Mo	0.05	-2.02	W	0.05	-3.70
Mn	0.07	-3.61	Tc	0.00	-1.88	Re	0.00	-1.38
Fe	0.13	-2.78	Ru	0.00	-1.74	Os	0.00	-1.14
Co	0.13	-2.60	Rh	0.13	-1.69	Ir	0.14	-1.27
Ni	0.00	-2.57	Pd	0.00	-1.41	Pt	0.00	-1.58
Cu	0.08	-1.20	Ag	0.00	0.60	Au	0.00	-0.06
Zn	0.00	-1.53	Cd	0.00	-0.13	Hg	0.00	1.45

Table S5. Spin moments and formation energies of FeM'-II DACs.

**3d TM**	**Fe spin moment (μB)**	**Formation Energy (eV)**	**4d TM**	**Fe spin moment (μB)**	**Formation Energy (eV)**	**5d TM**	**Fe spin moment (μB)**	**Formation Energy (eV)**
Sc	1.52	-3.99	Y	1.47	-3.80			
Ti	1.65	-2.92	Zr	1.54	-2.55	Hf	1.50	-2.57
V	1.80	-2.08	Nb	1.62	-1.03	Ta	1.67	-0.72
Cr	1.87	-2.61	Mo	1.81	0.70	W	1.71	-0.59
Mn	1.79	-2.90	Tc	1.85	0.25	Re	1.82	1.35
Fe	1.85	-2.35	Ru	1.85	-0.14	Os	1.84	0.68
Co	1.85	-2.54	Rh	1.86	-1.35	Ir	1.86	-0.88
Ni	1.87	-2.86	Pd	1.86	-1.80	Pt	1.86	-1.96
Cu	1.89	-1.56	Ag	1.55	0.89	Au	1.36	0.20
Zn	1.87	-2.00	Cd	1.87	0.93	Hg	1.64	4.63

Table S6. Spin moments and formation energies of CoM'-II DACs. The screening results for OER/ORR catalysts are highlighted.

**3d TM**	**Co spin moment**	**Formation Energy**	**4d TM**	**Co spin moment**	**Formation Energy**	**5d TM**	**Co spin moment**	**Formation Energy**

	(μB)	(eV)		(μB)	(eV)		(μB)	(eV)
Sc	0.00	-4.42	Y	0.00	-4.25			
Ti	0.16	-3.25	Zr	0.03	-2.94	Hf	0.04	-2.95
**V**	**0.58**	**-2.61**	Nb	0.05	-1.38	**Ta**	**0.38**	**-1.00**
**Cr**	**0.73**	**-2.82**	Mo	0.60	0.40	**W**	**0.55**	**-0.93**
**Mn**	**0.64**	**-3.14**	Tc	0.71	-0.01	Re	0.64	1.09
**Fe**	**0.75**	**-2.53**	**Ru**	**0.76**	**-0.35**	Os	0.71	0.54
**Co**	**0.75**	**-2.70**	**Rh**	**0.73**	**-1.55**	**Ir**	**0.75**	**-1.07**
**Ni**	**0.78**	**-3.00**	**Pd**	**0.77**	**-1.98**	**Pt**	**0.79**	**-2.14**
**Cu**	**0.64**	**-1.54**	Ag	0.16	0.46	Au	0.00	-0.22
Zn	0.78	-2.21	Cd	0.79	0.67	Hg	0.52	4.24

Table S7. Spin moments and formation energies of NiM'-II.

3d TM	Ni spin moment (μB)	Formation Energy (eV)	4d TM	Ni spin moment (μB)	Formation Energy (eV)	5d TM	Ni spin moment (μB)	Formation Energy (eV)
Sc	0.00	-3.72	Y	0.00	-3.53			
Ti	0.01	-2.72	Zr	0.01	-2.31	Hf	0.01	-2.28
V	0.02	-2.00	Nb	0.01	-0.75	Ta	0.00	-0.43
Cr	0.01	-2.53	Mo	0.01	0.78	W	0.02	-0.47
Mn	0.01	-2.75	Tc	0.00	0.31	Re	0.00	1.54
Fe	0.01	-2.23	Ru	0.00	-0.07	Os	0.00	0.78
Co	0.00	-2.40	Rh	0.00	-1.26	Ir	0.00	-0.78
Ni	0.00	-2.72	Pd	0.00	-1.71	Pt	0.00	-1.86
Cu	0.03	-1.46	Ag	0.13	0.97	Au	0.00	0.64
Zn	0.00	-1.96	Cd	0.00	0.89	Hg	0.00	4.65

Table S8. Spin moments and formation energies of FeM'-III DACs.

3d	Fe spin	Formation	4d	Fe spin	Formation	5d	Fe spin	Formation

TM	moment (μB)	Energy (eV)	TM	moment (μB)	Energy (eV)	TM	moment (μB)	Energy (eV)
Sc	1.65	-3.75	Y	1.64	-3.61			
Ti	1.77	-2.42	Zr	1.62	-2.32	Hf	1.64	-2.31
V	1.89	-1.98	Nb	1.77	-0.27	Ta	1.78	-0.09
Cr	1.89	-2.46	Mo	1.89	0.51	W	1.88	-0.69
Mn	1.87	-2.56	Tc	1.88	0.21	Re	1.88	1.32
Fe	1.89	-1.89	Ru	1.88	-0.08	Os	1.88	0.80
Co	1.90	-2.06	Rh	1.89	-1.34	Ir	1.89	-0.81
Ni	1.89	-2.31	Pd	1.89	-1.56	Pt	1.89	-1.71
Cu	1.89	-1.04	Ag	1.91	0.88	Au	1.62	0.46
Zn	1.90	-1.85	Cd	1.88	0.57	Hg	1.86	4.26

Table S9. Spin moments and formation energies of CoM'-III DACs. The screening results for OER/ORR catalysts are highlighted.

3d TM	Co spin moment (μB)	Formation Energy (eV)	4d TM	Co spin moment (μB)	Formation Energy (eV)	5d TM	Co spin moment (μB)	Formation Energy (eV)
**Sc**	**0.40**	**-3.08**	Y	0.00	-1.37			
**Ti**	**0.55**	**-2.54**	Zr	0.33	-2.46	Hf	0.34	-2.45
**V**	**0.64**	**-2.01**	**Nb**	**0.55**	**-0.35**	**Ta**	**0.58**	**-0.21**
**Cr**	**0.65**	**-2.51**	Mo	0.65	0.44	**W**	**0.68**	**-0.75**
**Mn**	**0.59**	**-2.59**	Tc	0.61	0.21	Re	0.62	1.49
**Fe**	**0.59**	**-1.90**	Ru	0.70	0.12	Os	0.02	0.84
**Co**	**0.71**	**-1.96**	**Rh**	**0.73**	**-1.31**	**Ir**	**0.72**	**-0.77**
**Ni**	**0.59**	**-2.30**	**Pd**	**0.59**	**-1.58**	**Pt**	**0.60**	**-1.72**
**Cu**	**0.62**	**-1.07**	Ag	0.84	0.81	Au	0.36	0.32
**Zn**	**0.59**	**-1.91**	Cd	0.65	0.47	Hg	0.58	4.20

Table S10. Spin moments and formation energies of NiM'-III DACs.

3d TM	Ni spin moment (μB)	Formation Energy (eV)	4d TM	Ni spin moment (μB)	Formation Energy (eV)	5d TM	Ni spin moment (μB)	Formation Energy (eV)
Sc	0.00	-4.19	Y	0.00	-4.07			
Ti	0.00	-3.19	Zr	0.00	-2.85	Hf	0.00	-2.77
V	0.00	-2.45	Nb	0.00	-1.34	Ta	0.00	-0.69
Cr	0.00	-2.69	Mo	0.01	0.49	W	0.01	-0.68
Mn	0.00	-2.82	Tc	0.00	0.18	Re	0.00	1.04
Fe	0.00	-2.22	Ru	0.00	-0.22	Os	0.00	0.71
Co	0.01	-2.47	Rh	0.00	-1.57	Ir	0.00	-0.99
Ni	0.00	-2.69	Pd	0.00	-1.73	Pt	0.00	-1.83
Cu	0.00	-1.39	Ag	0.00	0.81	Au	0.00	0.32
Zn	0.00	-2.15	Cd	0.00	0.44	Hg	0.00	3.83

Table S11. Spin moments on Fe/Co/Ni sites, formation energies, DFT-calculated oxygenate intermediate binding energies, and OER activity of screened DACs for OER catalysis.

DACs	Spin moment of M (μB)	Formation energy (eV)	ΔE_{HO*} (eV)	ΔE_{O*} (eV)	ΔE_{HOO*} (eV)	U_{RHE} @ 10 mAcm^{-2} (V)
CoTa-II	0.38	-1.00	0.94	2.83	3.95	1.606
CoW-II	0.49	-0.93	0.88	2.71	3.91	1.600
CoSc-III	0.40	-3.08	0.87	2.55	3.90	1.603
CoTi-III	0.55	-2.54	0.82	2.43	3.86	1.603
CoNb-III	0.55	-0.35	0.85	2.50	3.87	1.612

Table S12. Spin moments on Fe/Co/Ni sites, formation energies, DFT-calculated oxygenate intermediate binding energies, and ORR activity of screened DACs for ORR catalysis.

DACs	Spin moment of M (μB)	Formation energy (eV)	ΔE_{HO*} (eV)	ΔE_{O*} (eV)	ΔE_{HOO*} (eV)	TOF @ 0.9 V_{RHE} (s^{-1})
FeCr-I	1.20	-3.45	0.47	1.85	3.48	-0.974
FeMn-I	1.10	-3.68	0.52	1.97	3.55	-0.782
FeRe-I	1.12	-1.52	0.61	2.15	3.55	-0.788
CoCu-II	0.79	-1.54	0.67	2.28	3.59	-0.867
CoPt-II	0.79	-2.14	0.67	2.37	3.59	-0.872

Table S13. Spin moments on Fe/Co/Ni sites, formation energies, DFT-calculated oxygenate intermediate binding energies, and ORR/OER activities of screened DACs for bifunctional oxygen catalysis. The promising bifunctional catalysts are highlighted.

DACs	Spin moment of M (μB)	Formation energy (eV)	ΔE_{HO*} (eV)	ΔE_{O*} (eV)	ΔE_{HOO*} (eV)	TOF @ 0.9 V_{RHE} (s^{-1})	U_{RHE} @ 10 $mAcm^{-2}$ (V)
**CoNi-I**	**0.66**	**-2.45**	**0.86**	**2.63**	**3.86**	**-1.447**	**1.608**
**CoCu-I**	**0.69**	**-1.05**	**0.79**	**2.54**	**3.86**	**-1.174**	**1.659**
**CoZn-I**	**0.70**	**-1.21**	**0.74**	**2.44**	**3.76**	**-1.040**	**1.695**
**CoPd-I**	**0.64**	**-1.43**	**0.83**	**2.62**	**3.85**	**-1.329**	**1.627**
**CoPt-I**	**0.65**	**-1.59**	**0.84**	**2.63**	**3.83**	**-1.358**	**1.621**
**CoV-II**	**0.58**	**-2.61**	**0.77**	**2.47**	**3.69**	**-1.217**	**1.650**
CoCr-II	0.73	-2.82	0.68	2.36	3.62	-0.888	1.748
**CoMn-II**	**0.70**	**-3.14**	**0.76**	**2.48**	**3.72**	**-1.098**	**1.679**
CoFe-II	0.75	-2.53	0.67	2.42	3.62	-0.874	1.754
CoCo-II	0.75	-2.69	0.68	2.25	3.61	-0.893	1.746
CoNi-II	0.78	-3.00	0.67	2.31	3.58	-0.866	1.757
CoZn-II	0.78	-2.20	0.67	2.41	3.61	-0.875	1.753
CoRu-II	0.76	-0.35	0.73	2.33	3.76	-0.919	1.735
CoRh-II	0.73	-1.55	0.69	2.31	3.71	-0.836	1.775

CoPd-II	0.77	-1.98	0.65	2.31	3.60	-0.911	1.738
CoIr-II	0.76	-1.07	0.69	2.33	3.71	-0.872	1.755
**CoV-III**	**0.64**	**-2.01**	**0.77**	**2.43**	**3.75**	**-1.136**	**1.669**
**CoCr-III**	**0.65**	**-2.51**	**0.79**	**2.45**	**3.92**	**-1.185**	**1.657**
CoMn-III	0.59	-2.59	0.73	2.3	3.87	-1.006	1.705
CoFe-III	0.59	-1.90	0.73	2.34	3.86	-1.000	1.707
**CoCo-III**	**0.71**	**-1.96**	**0.75**	**2.36**	**3.74**	**-1.072**	**1.686**
CoNi-III	0.59	-2.30	0.72	2.28	3.83	-0.988	1.711
**CoCu-III**	**0.62**	**-1.07**	**0.73**	**2.41**	**3.83**	**-1.019**	**1.701**
**CoZn-III**	**0.59**	**-1.91**	**0.75**	**2.41**	**3.83**	**-1.136**	**1.669**
CoRh-III	0.73	-1.31	0.67	2.31	3.70	-0.876	1.753
**CoPd-II**	**0.59**	**-1.58**	**0.75**	**2.36**	**3.85**	**-1.065**	**1.688**
**CoTa-III**	**0.58**	**-0.21**	**0.83**	**2.49**	**3.90**	**-1.335**	**1.626**
CoW-III	0.68	-0.75	0.77	2.35	3.79	-0.974	1.716
**CoIr-III**	**0.72**	**-0.77**	**0.74**	**2.40**	**3.85**	**-1.045**	**1.694**
**CoPt-III**	**0.60**	**-1.72**	**0.74**	**2.36**	**3.86**	**-1.045**	**1.694**

Referecences

(1) Zhang, D.; Wang, Z.; Liu, F.; Yi, P.; Peng, L.; Chen, Y.; Wei, L.; Li, H. Unraveling the pH-dependent oxygen reduction performance on single-atom catalysts: from single- to dual-sabatier optima. *J. Am. Chem. Soc.* **2024,** *146*, 3210-3219.

(2) Hansen, H. A.; Viswanathan, V.; Nørskov, J. K. Unifying kinetic and thermodynamic analysis of $2e^-$ and $4e^-$ reduction of oxygen on metal surfaces. *J. Phys. Chem. C* **2014,** *118*, 6706-6718.

(3) Kelly, S. R.; Kirk, C.; Chan, K.; Nørskov, J. K. Electric field effects in oxygen reduction kinetics: rationalizing pH dependence at the Pt(111), Au(111), and Au(100) electrodes. *J. Phys. Chem. C* **2020,** *124*, 14581-14591.

(4) Dickens, C. F.; Kirk, C.; Nørskov, J. K. Insights into the electrochemical oxygen evolution reaction with ab Initio calculations and microkinetic modeling: beyond the limiting potential volcano. *J. Phys. Chem. C* **2019,** *123*, 18960-18977.

(5) Zhang, D.; Li, H. Digital catalysis platform (DigCat): A gateway to big data and AI-powered innovations in catalysis. ChemRxiv **2024**.

(6) Cui, J.; Zhang, D.; Liu, Z.; Li, C.; Zhang, T.; Yin, S.; Song, Y.; Li, H.; Li, H.; Li, C., Carbon-anchoring synthesis of Pt_1Ni_1@Pt/C core-shell catalysts for stable oxygen reduction reaction. *Nat. Commun.* **2024,** *15*, 9458.

(7) Bender, J. T.; Sanspeur, R. Y.; Bueno Ponce, N.; Valles, A. E.; Uvodich, A. K.; Milliron, D. J.; Kitchin, J. R.; Resasco, J., How Electrolyte pH Affects the Oxygen Reduction Reaction. *J. Am. Chem. Soc.* **2025,** *147*, 37819-37832.

(8) Li, H.; Kelly, S.; Guevarra, D.; Wang, Z.; Wang, Y.; Haber, J. A.; Anand, M.; Gunasooriya, G. T. K. K.; Abraham, C. S.; Vijay, S.; Gregoire, J. M.; Nørskov, J. K. Analysis of the limitations in the oxygen reduction activity of transition metal oxide surfaces. *Nat. Catal.* **2021,** *4*, 463-468.

(9) Zhang, D.; Hirai, Y.; Nakamura, K.; Ito, K.; Matsuo, Y.; Ishibashi, K.; Hashimoto, Y.; Yabu, H.; Li, H. Benchmarking pH-field coupled microkinetic modeling against oxygen reduction in large-scale Fe-azaphthalocyanine catalysts. *Chem. Sci.* **2024,** *15*, 5123-5132.

(10) Zhang, D.; She, F.; Chen, J.; Wei, L.; Li, H. Why do weak-binding M-N-C single-atom catalysts possess anomalously high oxygen reduction activity? *J. Am. Chem. Soc.* **2025,** *147*, 6076-6086.
